# Immune Responses to Non-Tumor Antigens in the Central Nervous System

**DOI:** 10.3389/fonc.2014.00328

**Published:** 2014-11-13

**Authors:** Amanda K. Huber, Patrick C. Duncker, David N. Irani

**Affiliations:** ^1^Department of Neurology, University of Michigan Medical School, Ann Arbor, MI, USA

**Keywords:** neuroimmunology, non-tumor antigens, glial cells, CNS autoimmunity, blood–brain barrier, CNS infections

## Abstract

The central nervous system (CNS), once viewed as an immune-privileged site protected by the blood–brain barrier (BBB), is now known to be a dynamic immunological environment through which immune cells migrate to prevent and respond to events such as localized infection. During these responses, endogenous glial cells, including astrocytes and microglia, become highly reactive and may secrete inflammatory mediators that regulate BBB permeability and recruit additional circulating immune cells. Here, we discuss the various roles played by astrocytes, microglia, and infiltrating immune cells during host immunity to non-tumor antigens in the CNS, focusing first on bacterial and viral infections, and then turning to responses directed against self-antigens in the setting of CNS autoimmunity.

## Introduction

The central nervous system (CNS) was previously viewed as an immune-privileged area, fully isolated from the immune system by the blood–brain barrier (BBB). In early studies, Ehrlich reported that while various organs were strongly stained following intravenous, intra-arterial, or subcutaneous injection of intravital dyes, the brain was only weakly stained or not at all (1). Other studies found that tissue grafts were not rejected when implanted into the brains of test animals (2), leading to the idea that the CNS was fully “immune-privileged.” This viewpoint had to be altered, however, after it was discovered that a graft within the CNS could be rejected if a second graft was placed subcutaneously into the same animal (3). This finding clearly demonstrated that foreign antigens are recognized in the CNS if peripheral priming occurs (3). It is now accepted that the BBB is a dynamic, interactive, and regulatory tissue interface that allows bi-directional communication between the CNS and the immune system (4, 5).

The BBB, formed by complex interactions between capillary endothelial cells (ECs), astrocyte end-feet, pericytes, and microglia (6, 7), is the largest and most stringent barrier that impedes the paracellular movement of ions, solutes, proteins, water, and leukocytes into the CNS (8). However, the BBB can also be influenced by peripheral immune events, creating what has now come to be known as the neuro-immune axis (4, 9, 10). The neuro-immune axis is not only responsible for establishing the blood–CNS barrier at baseline, but it also regulates communication between the CNS and the immune system during pathological conditions such as viral or bacterial infections, ischemia, or inflammatory autoimmune disorders such as multiple sclerosis (MS) (11). It achieves this state by responding to secreted factors from both immune and CNS cells, as well as by regulating the exchange of chemokines, cytokines, and immune cells between the blood and the CNS (4, 9, 10). Therefore, the original concept of the BBB being a purely anatomical barrier has now shifted to one where the BBB is considered a highly reactive interface controlled by signals from ECs, glial cells, pericytes, and neurons in the CNS, as well as from immune responses in the periphery (12–21).

### Structural characteristics of the BBB

The BBB is composed of capillary ECs ensheathed by astrocyte end-feet, pericytes, and microglia (6, 7). Astrocyte end-feet completely surround the abluminal surface of brain capillaries forming a layer known as the glial limitans, but direct contact with EC is inhibited by a dense basement membrane (22). While astrocytes are necessary to maintain BBB integrity by secreting factors that alter barrier permeability (6, 23), they are not actually required to form the BBB, which develops even before these astrocytic processes are present (24–26). Astrocytes control blood flow to the CNS by regulating vascular tone through fluctuating calcium currents (27). Pericytes are essential to barrier formation, as the BBB is compromised in pericyte-deficient mice (28, 29). These cells regulate gene expression in EC and induce the polarization of astrocyte end-feet (28). Microglia play a role at the BBB by regulating substrate transport across EC and by linking the brain to systemic immune activity (30).

Blood–brain barrier EC forms a highly sophisticated barrier via a network of tight junctions (TJ) and adherens junctions (AJ) (8, 31, 32). The EC of the CNS are unique in that the TJ restrict the paracellular passage of solutes, have no pinocytic activity, and have few if any fenestrations (33–39). This causes the BBB to have high endothelial electrical resistance (40, 41), some 50–100 times higher than peripheral microvessels (42–44). The TJ are composed of a parallel network of intramembranous protein strands, composed of claudins, occludin, and zonula occludin (ZO) proteins (37). Claudins, specifically claudin-3, -4, and -12, compose the TJ backbone (45–47). Occludin is not required for TJ formation (48); instead, it plays a role in “sealing” the junction thereby increasing electrical resistance (49, 50). CNS microvessel TJ are also abundant in ZO-2, and to a lesser extent, ZO-1, that are cytoplasmic accessory proteins that serve to anchor the transmembrane proteins of the TJ to the actin cytoskeleton of the ECs (51, 52).

The choroid plexus (CP) is a villous structure located on the roof of the four cerebral ventricles where cerebrospinal fluid (CSF) is actively secreted. The CP is highly vascular and contains the blood–CSF barrier (BCSFB) (51). Unlike the BBB, however, the BCSFB arises from cuboidal choroid plexus epithelial cells (CPE) with a very different TJ structure. The CPE express ZO-1 and ZO-2 in different amounts (51), and have a different claudin signature, expressing claudin-1, -2, -3, and -11 (51, 53, 54). Furthermore, capillaries within the CP villi are fenestrated (51, 55, 56), reflected by a much lower endothelial electrical resistance than the BBB (57). For these reasons, the BBB is considered more of an absolute barrier, while the BCSFB may be where most normal immune surveillance of the CNS occurs (58).

### Immune surveillance and infiltration of the CNS

It is now accepted there is a constant need for immune surveillance of the normal CNS as part of host defense (11, 59, 60), with mechanisms present that simultaneously keep excessive inflammation in check (61). To assist in maintaining this control, the healthy CNS is relatively devoid of antigen-presenting cells (APC), lacks constitutive human leukocyte antigen (HLA) class I and II protein expression on parenchymal cells, and does not maintain typical lymphatic vessels (11). CD4+ T cells, having first encountered antigens in deep cervical lymph nodes (62), carry out routine surveillance of the CNS by searching for their cognate antigens presented by macrophages in the CSF (11, 63). Resting lymphocytes fail to enter the CNS (64), while activated T cells of all specificities can traverse the BBB and/or BCSFB (65). Those cells that do not encounter their cognate antigen within a few hours then circulate out of the CNS (66, 67).

The first steps of pathogenic neuroinflammation involve changes at the BBB, including increased production of chemokines and up-regulation of adhesion molecules by the EC resulting in leukocytes traversing the BBB and accumulating in the perivascular space of post-capillary venules (11, 68). Even during these early events, however, cellular recruitment remains tightly controlled as parenchymal lymphocytes express a unique adhesion molecule profile, different from peripheral T cells (69–71). Once in the perivascular space, T cells encounter the glial limitans as well as astrocytes that express and release factors that induce apoptosis (72), inhibit proliferation (72), induce differentiation into a regulatory (Treg) phenotype (73). Microglia and neurons also assist in controlling neuroinflammation. Microglia do so by expressing a homolog of the co-stimulatory molecule B7, programed death protein (PD)-1, which negatively regulates T cell activation and cytokine production (74). Neurons secrete transforming growth factor (TGF)-β, exert cell contact-dependent effects that support the conversion of CD4 T cells to Tregs, and can be induced to express the PD-1 ligand, PD-L1 (75). Thus, while the BBB is not the impenetrable barrier it was once thought to be, CD4+ T cell surveillance of the CNS is still a tightly controlled process.

## Host Immune Responses to Bacterial Infections of the CNS

**Figure 1:**
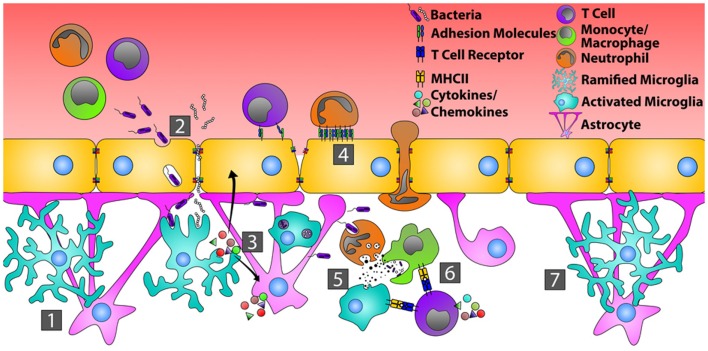
**Orchestration of the immune response during bacterial infection of the CNS**. In the quiescent CNS (1), bacteria typically gain entry by transcytosis across the endothelial cells of the BBB, or by passing in between endothelial cells where tight junctions have been disrupted (2). Common bacterial motifs (PAMPs) are recognized by pattern recognition receptors (PRRs) on microglia and astrocytes resulting in their activation. This causes changes in glial cell morphology, enhanced production of inflammatory mediators that recruit neutrophils, monocytes, and T cells, and increased endothelial cell expression of adhesion molecules, including ICAM-1 and VCAM-1 (3). Circulating neutrophils, monocytes, and T cells then bind and extravasate into the infected CNS (4). Neutrophils directly phagocytize and kill bacteria through the release of defensins, lytic enzymes, and anti-microbial peptides (5). Neutrophils also produce MMPs, IL-6, IL-8, CXCL9, and CXCL10 that further open the BBB and shift the chemotactic profile toward the recruitment of T cells. Bacterial antigens are presented to T cells by microglia and/or infiltrating monocytes, transitioning from innate immunity toward an adaptive immune response (6). Resolution of bacterial infection returns tight junctions to normal and microglia and astrocytes to a resting state (7).

Bacterial infections of the CNS are rare, but often life threatening, events (76). Excluding direct inoculation following CNS trauma, bacteria typically gain CNS entry following hematogenous dissemination from distant sites (lungs and heart valves) or by direct extension from parameningeal foci of infection (inner ear and sinuses). Penetration of the BBB may occur via three potential mechanisms: (1) direct destruction of capillary ECs (77, 78), (2) disruption of intercellular TJ and migration in between ECs (79), and (3) transcytosis via intracellular vesicles directly through ECs (80). Once inside, numerous innate immune receptors and pathways are activated (Figure 1).

### Microglia

Analogous to peripheral tissues, resident CNS immune cells known as microglia bear a wide range of innate immune receptors. Common bacterial motifs, referred to as pathogen associated molecular patterns (PAMP), are recognized by cognate pattern recognition receptors (PRR), including Toll-like receptors (TLR), on the surface and in the cytoplasm of microglia, and to a lesser extent, on astrocytes (81–83). Microglial activation, triggered either by intact bacteria or bacterial cell wall antigens (84, 85), results in rapid changes in cellular morphology *in vivo* (86). Similar to tissue resident macrophages found in the periphery, microglia can phagocytize bacteria and present bacterial antigens via HLA to infiltrating CD4 T cells *in vivo* (84, 87, 88). These cells also rapidly produce pro-inflammatory cytokines and chemokines that recruit peripheral leukocytes to the area of infection and activate astrocytes. For example, during both experimental *Streptococcus pneumoniae* and *Staphylococcus aureus* infections of the CNS, microglia produce tumor necrosis factor (TNF)-α, interleukin (IL)-6, IL-12, C-X-C motif ligand (CXCL)1, CXCL2, C-C motif ligand (CCL)2, CCL3, and CCL5 *ex vivo*, mediators that recruit neutrophils (CXCL1 and CXCL2), monocytes (CCL2 and CCL3), and T cells (CCL5) (84, 85, 89–91). These activated microglia also secrete matrix metalloproteinases (MMP) that enhance BBB breakdown and facilitate additional leukocyte extravasation into the CNS (92). Finally, microglia can have direct bactericidal activity, being capable of producing reactive oxygen species (ROS), reactive nitrogen intermediates, and other proteases that kill bacteria *in vivo* (93–96).

### Astrocytes

Microglia partner with astrocytes to eliminate infection as quickly as possible in order to minimize neuronal damage (86, 97, 98). In the normal CNS, astrocytes contribute to gap junction stability of the BBB (99). Their release of pro-inflammatory mediators such as IL-1β (100, 101), nitric oxide (102), TGF-β (103), and MMPs (92) *in vitro* suggest these cells may compromise BBB integrity in the setting of bacterial infection. Astrocytes are activated by bacterial PAMP or mediators produced by microglia; this changes their morphology and further triggers their release of innate inflammatory mediators both *in vitro* and *in vivo*. These mediators can include complement proteins, IL-1β, IL-6, and the chemokines, CCL2, CCL3, CXCL1, and CXCL10 (104–111), which further help recruit neutrophils, monocytes, and T cells. In response to interferon (IFN)-γ, TNF-α, and/or IL-1β, astrocytes also up-regulate the cell surface adhesion molecules, intercellular adhesion molecule (ICAM)-1, and vascular cell adhesion molecule (VCAM)-1 *in vitro* (112–116), which would enhance the infiltration of monocytes and T cells into the CNS *in vivo*.

### Neutrophils

As in the periphery (117, 118), neutrophils are one of the primary lines of host defense during CNS bacterial infections (112, 119, 120). Studies in knockout mice show that the main chemokines driving neutrophil recruitment to the CNS are the C-X-C motif receptor (CXCR)-2 ligands, CXCL1 and CXCL2 (121). Furthermore, CSF samples from patients with bacterial meningitis show elevated levels of neutrophil attracting chemokines compared to controls (122, 123). Neutrophils, like microglia, respond to PAMP through various TLR, and are activated by cytokines such as TNF-α and IFN-γ *in vitro* (124). Neutrophils activated in the periphery up-regulate adhesion molecules that enhance their migration into tissues (125), while BBB EC express E-selectin and P-selectin during CNS bacterial infection (126), suggesting a mechanism that allows for the migration of neutrophils during these infections. Once neutrophils recognize a bacterial pathogen, they can directly phagocytize these organisms (127), as well as release MMP, defensins, lytic enzymes, and anti-microbial peptides that aid in clearing the infection (128). The inflammatory cytokine, TNF-α, induces neutrophils to produce IL-6, IL-8, CXCL9, and CXCL10 *in vivo* (129, 130), thereby shifting the chemotactic profile toward the recruitment of T cells and driving the adaptive immune response.

### T cells

Adaptive immune responses are important in fighting CNS bacterial infections (131). During bacterial meningitis, T cell production of IFN-γ leads to the generation of chemokines that preferentially recruit monocytes and more T cells (132), supporting the transition from an innate to an adaptive immune response. Furthermore, IFN-γ, potentially made locally by T cells, increases the antigen-presenting capacity of microglial cells *in vitro* via up-regulation of HLA class I and II molecules, the co-stimulatory molecules, B7-1 and B7-2, and CD40 (133, 134). Bacterial antigen presentation by microglia activates T cells (135), driving further T cell proliferation and greater production of IFN-γ.

## Host Immune Responses to Viral Infections of the CNS

Viruses use a variety of mechanisms to gain entry into the CNS. In the case of alphaherpesviruses (i.e., herpes simplex virus and varicella-zoster virus) and rabies virus, infection of peripheral nerves allows viral particles to travel by anterograde axonal transport into the CNS. Human immunodeficiency virus and human T cell leukemia virus-I enter the CNS parenchyma by infecting host immune cells in the periphery, and using them as “Trojan horses” to carry viral particles across the BBB. Finally, Epstein–Barr virus and West Nile virus directly infect the ECs of the BBB, resulting in barrier disruption and enhanced migration of immune cells into the parenchyma (136).

**Figure 2:**
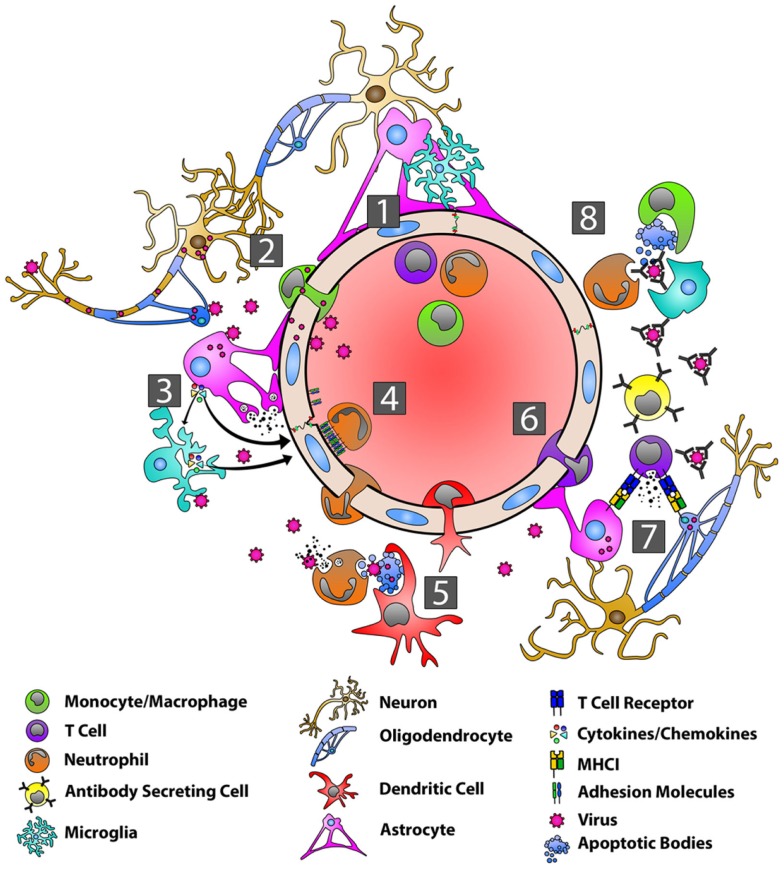
**Orchestration of the immune response during viral infection of the CNS**. With the BBB in a resting state (1), viruses can gain entry into the CNS by infecting peripheral nerves and traveling by anterograde axonal transport into the CNS, by infecting host immune cells in the periphery and using these cells as “Trojan horses” to carry them across the BBB, or by directly infecting BBB endothelial cells (2). Viral PAMPs then activate microglia, astrocytes, and oligodendrocytes (3). Microglia and astrocytes produce a range of anti-viral/pro-inflammatory cytokines, including type-I IFNs, IL-6, TNF-α, IL-12, IL-1α, and IL-1β (3). Astrocytes also produce MMP-3 and MMP-12 resulting in the up-regulation of adhesion molecules on endothelial cells (3). Interactions between adhesion molecules and neutrophils contribute to BBB breakdown via the production of MMP-9 and the disassembly of the tight junctions (4). DCs are seen in the CNS within several days and migrate to draining lymph nodes where they activate and expand virus-specific T cells (5). Chemokines produced by astrocytes are responsible for recruiting virus-specific CD4+ and CD8+ T cells as well as ASCs to the CNS (6). CD8+ T cells produce IFN-γ and lytic molecules, including granzyme B and perforin, to eliminate virus from astrocytes, while IFN-γ controls viral replication in oligodendrocytes (7). Virus-specific antibodies control virus replication in cells such as neurons via complement–independent, non-cytolytic mechanisms. These antibodies inhibit virus budding and replication, viral RNA transcription, and cell-to-cell virus spread.

Because viruses can infect microglia, astrocytes, oligodendrocytes, as well as terminally differentiated and non-renewable cells such as neurons, the ensuing immune response within the CNS must avoid extensive cytolytic damage of virus-infected target cells (137). In general, innate anti-viral immunity such as the generation of type-I IFN occurs very rapidly, while the adaptive immune response is slower because it must first develop in the periphery (138). Important components of adaptive anti-viral immunity involve IFN-γ production by T cells as well as the expansion and migration of virus-specific antibody secreting cells (ASC) (138, 139) (Figure 2).

### Microglia, astrocytes, and oligodendrocytes

During CNS viral infections, virus-specific PAMP activate individual TLR present on microglia, astrocytes, and oligodendrocytes. The former two cell populations, in particular, respond by producing anti-viral and pro-inflammatory mediators. During experimental mouse hepatitis virus (MHV) infection, astrocytes and microglia produce both type-I IFN (IFN-α and IFN-β), as well as IL-6, TNF-α, IL-12, IL-1α, and IL-1β *in vivo* (140–142). Furthermore, MHV infection triggers MMP-3 and MMP-12 release from astrocytes and oligodendrocytes (142), which along with IL-6 and the up-regulation of adhesion molecules on cerebrovascular endothelium, enhance cellular migration across the BBB (143). Astrocytes produce CXCL10, CXCL11, and CCL5 *in vivo* that recruit virus-specific CD4+ and CD8+ T cells (144–146), as well as ASC (147, 148), to the CNS to promote viral clearance. CXCL9 production from microglia is dependent on IFN-γ, while CXCL10 and CXCL11 are up-regulated by type-I IFN and TNF-α (149–152).

### Myeloid lineage cells

Neutrophils and macrophages are recruited to the CNS following viral infection (153, 154). Thus far, macrophages appear to have more limited anti-viral activity in the CNS (155), but neutrophils contribute to the breakdown of the BBB by interacting with EC via adhesion molecules to promote the disassembly of tight junction complexes (156). Neutrophils also secrete MMP-9 that degrades the extracellular matrix and basal lamina of the BBB and further opens the BBB (157). This has been most clearly demonstrated in the MHV model, where depletion of MMP-9 inhibited lymphocyte infiltration into the CNS (157, 158). Dendritic cells (DC) are seen in the CNS within a few days after CNS viral infection. These cells rely on the chemokine CCL3 to migrate to cervical lymph nodes draining the CNS, where they prime virus-specific T cells (159).

### T cells

In the MHV model, virus-specific CD8+ T cells are detected in local lymph nodes prior to CNS infiltration and then accumulate in the CNS (160). Both CD4+ and CD8+ T cells are in part recruited to the CNS by the chemokines, CXCL9 and CXCL10, acting through their cognate receptor, CXCR3 (161–163). T cell expression of CCR2 and CCR5 likely contribute to CNS recruitment as well (164, 165). The role of CD4+ T cells in this setting is to support CD8+ T cell function via the production of IFN-γ (166). CD8+ T cells are the main anti-viral effector cells in the CNS during infection and are essential for clearing virus from glial cells (142, 167, 168). CD8+ T cells produce IFN-γ and lytic molecules, including granzyme B and perforin (169). These lytic molecules eliminate virally infected astrocytes (170), while IFN-γ serves to control viral replication in oligodendrocytes (171, 172). In both the MHV and Sindbis virus (SINV) encephalitis models, T cells promote B cell proliferation and differentiation (173, 174), in part by secreting the cytokines, IL-10 and IL-21 (175–177).

### B cells and anti-viral antibodies

Virus-specific ASC help control viruses in the CNS through potent complement-independent, non-cytolytic mechanisms (141, 178–183). These ASC arise either from ectopic lymphoid follicle-like structures within the CNS (152) or migrate from cervical lymph nodes where they have expanded and up-regulated CXCR3 and CXCR4 on their surface prior to entering the CNS (184). ASC recruitment to the CNS has been most extensively studied in the SINV encephalitis model. The initial ASC entering the CNS have an HLA class II positive, plasmablast-like phenotype, but these cells gradually lose HLA class II expression and acquire a more plasma cell-like phenotype (139, 141). Virus-specific antibodies function to neutralize both extracellular virus as well as virus particles budding from infected cell membranes. During SINV infection, antibodies that bind the E2 viral envelope glycoprotein inhibit virus replication (185) and prevent viral budding from infected neurons without actually killing target cells (182, 186). Similarly, during rabies virus infection, antibodies against the RV glycoprotein inhibit viral RNA transcription and prevent cell-to-cell viral spread (187). Antibodies can also trigger natural killer (NK) cells and macrophages to induce antibody dependent cell-mediated cytolysis of virally infected cells (152). Finally, in exchange for non-cytolytic viral clearance in the acute setting, virus-specific ASC must persist in the CNS long term to prevent viral reactivation at a later date since viral RNA is never fully eradicated from target tissues (139).

## Host Immune Responses to Self (Myelin) Antigens in the CNS

### Multiple sclerosis

Multiple sclerosis, an autoimmune disease characterized by infiltrating immune cells targeting myelin antigens in the CNS, is the most common cause of neurologic disability in persons younger than 40 years of age (188). Pathologically, MS lesions are characterized by focal inflammation, demyelination, and axonal damage (189). MS is a complex disease whose occurrence and progression are influenced by both genetic (190–192) and environmental (193, 194) risk factors. Evidence derived from both human genetic studies and a related mouse model, experimental autoimmune encephalomyelitis (EAE), suggest that encephalitogenic CD4+ T cells are primary initiators of disease. Genome-wide association studies show that MS risk alleles are confined to immune related genes governing antigen presentation as well as the proliferation and survival of T cells, including HLA class II (HLA-DRB1*1501), IL-2R, and IL-7R (190–192). Moreover, EAE in mice is induced by immunizing animals with various myelin peptides (195), or via the adoptive transfer of myelin-specific CD4+ T cells, resulting in a disease having some clinical and pathological similarities to human MS (196, 197). In MS patients, CD4+ T cells localize within CNS lesions present in the brain (198) and spinal cord (199), and elevated frequencies of myelin-reactive CD4+ T cells can be found in circulating the blood (200, 201). Although not described in detail here due to space constraints, many MS lesions also contain abundant CD8+ T cells whose specificity and role in disease pathogenesis remain poorly understood. Likewise, therapies targeted specifically at B cells have proven highly effective in MS patients, highlighting an emerging role for this cell type in both relapsing and progressive forms of disease.

### Role of CD4+ T cells in autoimmune inflammation of the CNS

During both MS and EAE, self-reactive T cells are likely activated in the periphery (189), where they undergo initial differentiation and expansion (124). Upon entry into the CNS, these cells are reactivated by myelin epitopes presented by an as of yet unidentified local DC (202, 203). Production of cytokines such as IFN-γ and TNF-α from activated CD4+ T cells results in local activation of resting microglia, leading to the up-regulation of HLA class I and II as well as co-stimulatory molecules (B7-1, B7-2, and CD40) on the surface of these cells (133, 134, 204, 205). These activated microglia are capable of serving as APC for infiltrating myelin-specific CD4+ T cells *in vivo* thus sustaining this pathogenic local T cell response (97). Production of cytokines, chemokines, and MMPs by microglia (206) facilitate local inflammation by causing BBB breakdown and recruiting more immune cells into the CNS. These include circulating monocytes capable of differentiating into inflammatory DC and macrophages upon tissue entry (207), culminating in demyelination (124). Furthermore, microglial production of IL-23 and IL-1β promotes granulocyte macrophage colony-stimulating factor (GM-CSF) secretion by CD4+ T cells (208). GM-CSF has been shown in EAE to promote CNS inflammation by mobilizing Ly6C^hi^ monocytes from the bone marrow into the periphery, thereby increasing the number of circulating monocytes available for recruitment to the CNS (207). GM-CSF can also increase HLA class II expression and pro-inflammatory cytokine production by microglia, macrophages, and DC *in vitro* (209, 210). IL-17 producing T cells have been detected within CNS lesions during both EAE and MS (211, 212). IL-17 promotes brain inflammation, inducing the production of pro-inflammatory cytokines, TNF-α, IL-6, and IL-1β most probably from astrocytes, microglia, or macrophages. It also stimulates the release of chemokines responsible for recruiting neutrophils to the CNS, particularly CXCL1 and CXCL2 (213, 214). Finally, IL-17 can disrupt TJ in the BBB, allowing further migration of CD4+ T cells to the CNS (212, 215).

### Role of glial cells in autoimmune inflammation of the CNS

#### Microglia

Microglia play important roles in augmenting CNS inflammation, demyelination, and neuronal damage in both EAE and MS (67, 216–218). Activation of microglia occurs rapidly following the induction of EAE and results in the release of cytokines, chemokines, ROS, and tissue-degrading MMP (206). One mediator, TNF-like weak inducer of apoptosis (TWEAK), triggers proliferation, angiogenesis, inflammation, is associated with extensive myelin loss, and induces astrocyte cell death during MS (219). IL-17 produced by microglia (220) worsens brain inflammation by stimulating GM-CSF production, as well as increasing IL-6, inflammatory proteins, nitric oxide, and adhesion molecule expression by macrophages. Moreover, expression of myeloperoxidase (MPO) and ROS by microglia results in direct myelin degradation and neuronal damage (216, 218). Paradoxically, microglia also can play a neuroprotective role during CNS autoimmunity. These cells can promote remyelination, protect neurons, and suppress the adaptive immune response within the CNS (221, 222). Within MS lesions, microglia and macrophages express the neurotrophic factors, nerve growth factor (NGF), and brain-derived neurotrophic factor (BDNF), supporting neuronal survival (220, 223, 224). Furthermore, microglia secrete the anti-inflammatory cytokines, IL-10 and TGF-β, and express the inhibitory receptor, PD-L1, responsible for inhibiting T cell proliferation and cytokine production (74, 225).

#### Astrocytes

Astrocytes are a major source of CCL2 and CXCL10 in the CNS, regulating the migration of monocytes into the brain (CCL2) and microglia into the lesion site (CXCL10) (111, 226–228). One study suggested these cells play a more prominent role in regulating the recruitment of peripheral monocytes into the CNS (229). CXCL12, a chemokine that induces the expression of CXCL8 and CCL2, is also expressed by astrocytes in MS lesions (230). CXCL12 can be cleaved by MMP-2, also expressed by astrocytes in MS and EAE, into a neurotoxic peptide that causes further neuronal damage (231). Similar to microglia, astrocytes also play a protective role during MS and EAE. Homeostatic astrocyte functions include buffering potassium, removing extracellular glutamate that can accumulate to toxic levels, adjusting water balance, and controlling synaptic activity and blood flow in the CNS (8). These cells are also able to produce neurotrophins and the anti-inflammatory cytokine, IL-10 (232).

## Conclusion

The vast complexity of cellular interconnections within the CNS, and the non-renewable nature of many neural cells, mandate that some local immune responses be tightly controlled while others (i.e., cytolytic ones) be excluded to the fullest extent possible. The BBB is a dynamic and highly regulated tissue interface that helps make the CNS a unique immunological environment. It responds to signals from both neurons and glial cells on one side while simultaneously being able to sample immunological events passing through intravascular compartments. Immune cells perform normal surveillance of the CNS by searching for antigens previously encountered in extraneural sites such as the deep cervical lymph nodes. Pathological conditions such as infections caused by viruses or bacteria elicit changes at the BBB, including the up-regulation of a unique subset of adhesion molecules as well as heightened release of chemokines by ECs. Mediators produced by astrocytes and microglia further increase BBB permeability and recruit additional circulating leukocytes into the CNS. The ensuing immune response must then be tightly controlled in order to avoid collateral tissue damage. As such, astrocytes and microglia maintain mechanisms to dampen inflammatory responses. In some settings, immune cells such as ASC persist long term within the CNS to prevent viral reactivation. When normal control mechanisms fail, neuroinflammatory diseases such as MS can result. For this reason alone, it is imperative that the complexity of immune reactions within the CNS be better understood.

## Conflict of Interest Statement

The authors declare that the research was conducted in the absence of any commercial or financial relationships that could be construed as a potential conflict of interest.
